# Consistency of Parental and Self-Reported Adolescent Wellbeing: Evidence From Developmental Language Disorder

**DOI:** 10.3389/fpsyg.2021.629577

**Published:** 2021-03-11

**Authors:** Sheila M. Gough Kenyon, Olympia Palikara, Rebecca M. Lucas

**Affiliations:** ^1^Department of Psychology, University of Roehampton, London, United Kingdom; ^2^Department of Education Studies, University of Warwick, Coventry, United Kingdom

**Keywords:** developmental language disorder, low language ability, wellbeing, parent report, adolescent report

## Abstract

Research on adolescent wellbeing in Developmental Language Disorder (DLD) has previously been examined through measures of parent (proxy) or self-reported wellbeing, but never has a study included both and enabled comparison between the two. The current study reports parent and self rated wellbeing of adolescents with DLD and Low Language (LL) ability, as well as their typically developing (TD) peers. It also examines consistency between raters and factors influencing correspondence. Adolescents aged 10–11 with DLD (*n* = 30), LL (*n* = 29) or TD (*n* = 48) were recruited from eight UK primary schools. A battery of standardized language, psychosocial and wellbeing assessments, including the KIDSCREEN-27 were administered. Adolescent ratings of wellbeing were similar across groups on three of the five wellbeing dimensions, but those with DLD had lower self-reported Autonomy and Parental Relations than their TD peers, and both the DLD and LL group had lower School Environment scores than their TD peers. By parental report, the DLD and LL group were considered to have lower wellbeing on all five wellbeing dimensions relative to their TD peers. Paired sample *t*-test analyses indicated a high level of variance between parent and adolescent reported wellbeing for multiple wellbeing domains, especially Psychological Wellbeing. Importantly, predictors of the level of agreement between parent and adolescent reported psychological wellbeing differed between groups: cognitive reappraisal and sociability predicted this level of agreement for adolescents with LL, while social competence predicted agreement in DLD and TD. This study emphasizes the necessity of allowing adolescents of all language abilities to report their own wellbeing, as their perspective does not align with that of their parents. It also highlights the importance of including the full spectrum of need when investigating the impact of language ability on consistency between proxy and self-reported wellbeing.

## Introduction

There has been a recent surge of interest in adolescent wellbeing; partly due to a recognition that health goes beyond physical aspects (Statham and Chase, 2010) and also because of links between increased wellbeing and heightened intra-personal functioning and social cohesion (Hatch et al., 2007; Aminzadeh et al., 2013; Ding et al., 2015; Conti-Ramsden et al., 2016). Research has gradually shifted from reliance on parent report toward including adolescent's perspectives of their own wellbeing (Statham and Chase, 2010; Mashford-Scott et al., 2012; Coales et al., 2019), potentially due to increasing evidence that parent and adolescent perspective may not always align (Barblett and Maloney, 2010). However, parent vs. adolescent report of wellbeing is under-explored for families with adolescents with neurodevelopmental disorders, and there is a particular paucity of information for adolescents with Developmental Language Disorder (DLD). DLD was formerly commonly referred to as Specific Language Impairment (SLI), but the lack of consensus in terminology and criteria regarding language disorder led to a change in definition (Bishop et al., 2017). Thus, the current study aimed to examine consistency between parent and adolescent ratings of wellbeing, and factors influencing correspondence, for adolescents with DLD, adolescents with Low Language (LL) Ability, and their Typically Developing (TD peers).

Wellbeing is multifaceted (Black et al., 2019), and includes emotional (happiness, confidence, non-depressive symptomatology), psychological (autonomy, problem solving, resilience, attentiveness), and social (positive interpersonal relationships, absence of conduct disorder) aspects. This multifarious construct is therefore difficult to define and measure (Axford, 2009; Morrow and Mayall, 2009). The current study allows for the different dimensions of wellbeing by employing the KIDSCREEN-27 (The KIDSCREEN Group Europe, 2006), enabling Physical Wellbeing, Psychological Wellbeing, Autonomy and Parent Relations, Social Support and Peers, and School Environment to be measured.

However, less equivocal is the importance of wellbeing. This has been highlighted both internationally and in the UK in recent policy documents. A recent green paper in the UK (Transforming Children and Young People's Mental Health Provision; Department of Health and Social Care and Department for Education, 2017) sets out the British government's aims in regards to mental health and wellbeing in adolescence. It outlined the role of schools in supporting the mental health and wellbeing of adolescents and made proposals to improve this provision. Responses have highlighted the dated material statistics were drawn from (with one in ten adolescents cited as experiencing mental health difficulty when more recent prevalence rates are two in five; Deighton et al., 2019) and argued that more urgent action than that proposed by the green paper was needed (Bush, 2018). Importantly, while this green paper makes reference to vulnerable groups including those with Autism Spectrum Disorder (ASD), eating, conduct and emotional disorders, it makes no mention of children or adolescents with language disorders. This is despite the fact that language disorders are the most prevalent type of special educational need, comprising 23% of the Special Educational Needs and Disabilities (SEND) register in schools in England (Department for Education, 2019).

### Levels of Wellbeing in Developmental Language Disorder

DLD is a neurodevelopmental disorder affecting 7.5% of children (Tomblin et al., 1997; Norbury et al., 2016) and persisting into adolescence and adulthood. Adolescents with DLD may present with varying profiles of impairment spanning language areas and modalities. These impairments may be receptive, expressive or mixed (American Psychiatric Association (APA), 2013; Bishop et al., 2017). Adolescents with low language (LL) ability also present with impairment(s) in language, but not to the degree necessary to warrant a diagnosis of DLD. In addition, adolescents with DLD and those with LL can have pervasive needs in non-language domains such as psychosocial functioning (Conti-Ramsden et al., 2013; Yew and O'Kearney, 2013; Forrest et al., 2020; Toseeb and St Clair, 2020) and emotional regulation skill (Fujiki et al., 2002) and report lower self-perceived health than their TD peers (Beitchman et al., 2014). Given such challenges it is likely that individuals with DLD would be at increased risk of poorer wellbeing than their TD peers.

One method of assessing wellbeing of school aged children and adolescents is parental completion of questionnaires, and studies using such methodology indicate poorer wellbeing for individuals with DLD. For example, Van Agt et al. (2011) found that 8 year olds with language disorders (both receptive and expressive) had lower quality of life, poorer emotional stability and poorer mental health than their TD peers according to parental report. Similarly, Eadie et al. (2018) found that children with DLD had lower parent-reported wellbeing at 9 years than their TD peers. Interestingly, they found that within their sample of children with DLD, the severity of language impairment did not significantly affect levels of parent-reported wellbeing. Language skill at age 7, for the entire cohort (including TD children), was found to be a key indicator of parent-reported wellbeing at age 9.

Evidence of reduced wellbeing is also present for older children and adolescents with DLD. Hubert-Dibon et al. (2016) found that children and adolescents with DLD aged 8–18 (mean age = 10.25) received lower parental ratings on all five dimensions of the KIDCREEN-27 (Physical Wellbeing, Psychological Wellbeing, Autonomy and Parent Relations, Social Support and Peers, and School Environment), relative to their TD peers. A key strength of this study is the use of the KIDSCREEN, which not only includes multiple dimensions, but also has high reliability and validity (Ravens-Sieberer et al., 2007). Whilst initially developed as a measure of health-related quality of life (HRQoL; a multidimensional construct covering physical, emotional, mental, social, and behavioral components of well-being and functioning; Ravens-Sieberer et al., 2014), it has frequently been used as a proxy measure of wellbeing (Ravens-Sieberer et al., 2010; Lloyd and Emerson, 2016).

However, while parental report of their child's wellbeing is a valuable source of information, children and adolescents with DLD are capable of reporting their own experiences (Owen et al., 2004; Palikara et al., 2009), and it must be noted that parent and their child/adolescent perceptions can differ (Sweeting, 2001; Hughes et al., 2009). Indeed, there are significant discrepancies between parent and child/adolescent report of wellbeing in populations with neurodevelopmental disorders, such as ASD. In these studies, self-reported wellbeing was significantly higher than parent rates of their child's wellbeing (Potvin et al., 2015; Egilson et al., 2017). Thus, although wellbeing may appear lower for individuals with DLD when assessed via parental report (cf. Van Agt et al., 2011; Hubert-Dibon et al., 2016; Eadie et al., 2018), perhaps it may be similar to TD peers when measured via self-report. It has been suggested that young people with DLD could be satisfied with less; for example, experiencing lower levels of dissatisfaction associated with low academic attainment than their TD peers (Durkin et al., 2009), or that support networks (e.g., family) may act as protective factors for wellbeing given the pervasive needs of this population (Johnson et al., 2010).

Indeed, early research conducted by Records et al. (1992) found that 29 young adults with DLD reported similar levels of wellbeing to their TD peers. This finding is consistent with more recent research with young adults, which has included considerably larger samples (e.g., Johnson et al., 2010; Conti-Ramsden et al., 2016). However, these studies considered wellbeing as a unitary construct, and it is important to remember that wellbeing is a multifaceted construct. Arkkila et al. (2009, 2011) asked 12–16 year olds and 8–11 year olds, respectively, to complete multiple dimensions assessments of HRQoL (Apajasalo et al., 1996a,b). They found that the participants with DLD did not indicate poorer wellbeing, although they did have poorer functioning in the dimensions of sleep and mental functioning. Similarly, Coales et al. (2019) used the multi-dimensional KIDSCREEN-52 (Ravens-Sieberer et al., 2005) to measure the wellbeing of children and adolescents aged 7–13 (mean = 10.39) with DLD. Wellbeing was largely commensurate with TD normative ranges, although lower scores for the participants with DLD were evident for the Moods and Emotions and the Social Acceptance/Bullying domains. Similar discrepancies, specifically in the domain of Psychological Wellbeing, have been discussed in a literature review (Feeney et al., 2012). This review is limited to seven studies due to the paucity of research in the field, yet includes evidence of lower levels of self and parent reported psychological wellbeing for children with language impairments relative to their TD peers (Markham and Dean, 2006; Barr et al., 2007; Markham et al., 2009) and also instances of self-reported levels of psychological wellbeing of children with DLD equivalent to their TD peers (Fekkes, 2000; Van Agt et al., 2005; Arkkila et al., 2011).

Thus, the extant literature on the wellbeing of individuals with DLD has provided equivocal results. Parental report indicates that children and adolescents with DLD may be more vulnerable to poorer wellbeing than their peers. In contrast, self-reports by children and adolescents with DLD are more positive, and indicate wellbeing largely commensurate with that of their TD peers. This suggests that there may be poor correspondence between parent and self-report of wellbeing for children and adolescents with DLD. Indeed, there is low consistency between tutor-rated and self-rated levels of adaptability and school problems in adolescents with DLD (Valera-Pozo et al., 2020). However, to date no study has directly compared parent and self-report of wellbeing for children and adolescents with DLD using the same measure, making definitive conclusions difficult. In addition, there is a lack of knowledge regarding factors influencing parental and child report consistency.

### Predictors of Consistency Between Parental and Self-Reported Rating of Wellbeing

There are multiple factors which may influence the degree of correspondence between parental and child ratings of wellbeing, including, but not limited to, child competencies such as language, social functioning and emotion processing. Children with stronger language skills may be able to engage with their parents in more complex conversations regarding their wellbeing, and therefore it could be predicted that there would be a positive relationship between the child's language skill and the degree of correspondence between self-reported and proxy-reported wellbeing. However, this has yet to be explored. In order to examine this fully it would be important to include a wide spectrum of language competence, rather than focusing only on clinically referred samples (McKean et al., 2017).

Parents may also be susceptible to halo effects (Gooch et al., 2017), in this case perceiving that language impairments and associated deficits in areas such as social and emotional functioning influence other areas, i.e., wellbeing. Based on this premise, it may be expected that social competence would be positively associated with parental and child correspondence. For TD children and adolescents, peer relationships are associated with wellbeing (Feeney et al., 2012; Holder and Coleman, 2015). Thus, parents of children with DLD may be aware of their child's limited friendships and expect this to impact their wellbeing. However, the child themselves may not be so aware of their deficits (cf. Andrés-Roqueta et al., 2016), thus they have less of an impact on their wellbeing. This has yet to be examined.

A similar argument could be applied to emotion processing competence, especially emotion regulation (i.e., utilizing strategies to manage emotional response), which can be impaired for those with DLD (Fujiki et al., 2002; Conti-Ramsden et al., 2019). Emotion regulation competency promotes wellbeing of TD adolescents (Verzeletti et al., 2016; Morrish et al., 2018). Wellbeing is greater when the individual is disposed toward cognitive reappraisal (i.e., modifying the impact of an emotional experience) and lower when there is a tendency toward emotional suppression (i.e., modifying the external response to an emotional experience). Thus, if parents of adolescents with DLD are aware that their child has poorer emotion regulation than their peers, they may anticipate poorer wellbeing. This is an important avenue for future research.

### The Current Study

This study aims to contribute to the limited extant body of work exploring the wellbeing of adolescents with DLD aged 10–11 (cf. Hubert-Dibon et al., 2016). More specifically, it will compare the wellbeing of adolescents with DLD to their TD peers, examine consistency of parent (proxy) reported and adolescent self-reported wellbeing, and explore predictors of the raters' consistency for the dimension of Psychological Wellbeing. This is the only domain measured in each of the seven studies included in Feeney et al.'s (2012) literature review of HRQoL in children with language difficulties so was included for comparative purposes.

In addition, the study will uniquely include a third group of children; those who have poorer language skills than expected for their age, but who do not meet the diagnostic criteria for DLD. No research has been conducted measuring wellbeing in children with LL, yet McKean et al. (2017) discuss the limitations arising from the focus on clinically referred samples when so little is known about the impact of low language on functioning and wellbeing. We know that while adolescents with LL are at a similar risk of negative outcomes due to language impairment as their peers with DLD (Conti-Ramsden et al., 2017; Gough Kenyon et al., 2018), their lack of diagnosis means no entitlement to the support that a child with a diagnosis would be entitled to. These adolescents may employ different coping strategies, thus resulting in communication with parents being affected by different factors. Recent research has shown language skill to be an important factor; Coales et al. (2019) found language ability to be a moderating variable controlling much of the variance between DLD and ASD differences in wellbeing. In order to inform appropriate preventive interventions and include adolescents with language difficulties in policy going forward, we must understand the impact of the full spectrum of language needs.

The study has three hypotheses: Firstly, it is predicted that levels of wellbeing will differ by group according to parental report, with lower levels of wellbeing for adolescents with DLD and LL ability than their TD peers (cf. Hubert-Dibon et al., 2016; Eadie et al., 2018). The predictions for self-reported wellbeing are less certain, but it likely that the groups may be similar for at least some, but likely not all, dimensions (cf. Records et al., 1992; Arkkila et al., 2009, 2011; Johnson et al., 2010; Conti-Ramsden et al., 2016; Coales et al., 2019). Secondly, it is hypothesized that parent and adolescent ratings of wellbeing will not strongly align for the TD, LL or DLD groups (cf. Potvin et al., 2015; Egilson et al., 2017). Thirdly, it is expected that predictors of consistency between parent and adolescent ratings of the factor Psychological Wellbeing will include social competence and sociability (cf. Feeney et al., 2012; Conti-Ramsden et al., 2013) and cognitive reappraisal (cf. Fujiki et al., 2002, 2004; Verzeletti et al., 2016; Morrish et al., 2018).

## Method

### Participants

Participants (*n* = 107) were recruited to this study from eight primary schools in the UK. All participants were in the final year of primary school and aged 10–11. The protocol for this study was approved by the Research Ethics Committee at University of Roehampton, London. Informed consent was obtained from participants (verbal consent) and parents, teachers, and headteachers (written consent).

Participants with DLD (*n* = 30) were on their school's SEND register (a list of children with additional difficulties who need extra help and support within the school). They had an identified primary need of “Language Disorder” or “Speech, Language and Communication Need,” with no additional sensory impairments, and were receiving specialist educational support (e.g., learning support teacher). Their DLD symptomatology was indicated by their teachers through completion of the Children's Communication Checklist 2 (CCC-2; Bishop, 2003b). All participants completed a battery of standardized language assessments to confirm group membership. These assessments were the “Recalling Sentences” subtest (measuring expressive and receptive narrative) and the “Word Classes” subtest (Receptive and Expressive; measuring vocabulary) of the Clinical Evaluation of Language Fundamentals-IV (UK; Semel et al., 2004), and the Test for Reception of Grammar 2 (TROG-2; Bishop, 2003a; measuring receptive grammar). All adolescents included in the DLD group obtained a score at or below 1.25SD below the population norm on both a receptive and an expressive language task. These standardized assessments report a score of below 1.25 SD to be indicative of impairment. Please see Table 1 for details of participant's sex, language skill and cognitive ability standard scores by group.

**Table 1:** Participant sex breakdown, language skill and cognitive ability standard scores by group.

**Variable**	**Developmental Language Disorder mean (SD) *n* = 30**	**Low Language mean (SD) *n* = 29**	**Typically Developing mean (SD) *n* = 48**	***Test statistics***
**Sex**				
Male	12	11	26	X^2^ (2, *N* = 107) = 2.48, *p* = 0.289, φ = 0.15
Female	18	18	22	
Chronological Age (Years)	10.82^a^ (0.26)	10.86^a^ (0.23)	10.84^a^ (0.23)	*F*_(2, 106)_ = 0.23, *p =* 0.796, $\eta_{\text{p}}^{\;2}$ = 0.01
WASI-II Matrix Reasoning (T-score)	41.97^a^ (9.68)	48.69^b^ (7.57)	54.17^c^ (9.22)	*F*_(2, 106)_ = 17.26, *p < * 0.001, $\eta_{\text{p}}^{\;2}$ = 0.25
**Language skill**				
CELF Recalling Sentences (Scaled score)	7.13^a^ (3.61)	9.14^b^ (2.23)	11.27^c^ (1.85)	*F*_(2, 106)_ = 24.70, *p < * 0.001, $\eta_{\text{p}}^{\;2}$ = 0.32
CELF Vocabulary Word Classes Receptive (Scaled score)	5.87^a^ (1.50)	9.38^b^ (2.04)	12.69^c^ (2.69)	*F*_(2, 106)_ = 86.45, *p* < 0.001, $\eta_{\text{p}}^{\;2}$ = 0.62
CELF Vocabulary Word Classes Expressive (Scaled score)	5.93^a^ (2.00)	10.66^b^ (1.65)	13.90^c^ (2.47)	*F*_(2, 106)_ = 127.17, *p* < 0.001, $\eta_{\text{p}}^{\;2}$ = 0.71
Test for Reception of Grammar (Standard score)	91.33^a^ (15.73)	92.76^a^ (16.78)	106.33^b^ (6.43)	*F*_(2, 106)_ = 16.65, *p* < 0.001, $\eta_{\text{p}}^{\;2}$ = 0.24

a,b,c *Values with the same superscript do not differ when p < 0.05*.

The LL group (*n* = 29) included those students who did not meet the criteria for DLD yet scored at or below 1.25SD on one of the language tasks. Teacher completion of the CCC-2 (Bishop, 2003b) indicated concerns as to their communicative ability. Thus, they exhibited lower language ability than their peers included in the TD group but did not score at or below 1.25SD below the population norm on both a receptive and an expressive language task, as per the DLD group.

The TD group (*n* = 48) included 40 adolescents who achieved scores within 2SD of the population norm on all language tasks and eight participants who achieved scores within 2SD of the population norm on three of the language tasks and above 2SD of the population norm on one of the language tasks. No members of the TD group had a history of language impairment.

The three groups, DLD, LL and TD, did not differ in sex nor chronological age. The DLD and LL groups had lower scores on the language measures than their TD peers, as was expected with their group status. They also had lower non-verbal ability (cf. Norbury et al., 2016), as assessed using the Matrix Reasoning subtest of the Wechsler Abbreviated Scale of Intelligence –Second Edition (WASI-II; Wechsler, 2011).

### Materials and Procedure

All adolescent measures were administered in two sessions per participant. These sessions were on sequential days where possible, with exceptional interruptions due to weekends or pupil absence. Participants were seen individually by the same researcher (author XX) in a quiet room at their school. The first session included the Matrix Reasoning subtest of the WASI-II (Wechsler, 2011), followed by the “Recalling Sentences” and “Word Classes” subtests of the CELF-IV (Semel et al., 2004), and then the TROG-2 (Bishop, 2003a). In the second session, the KIDSCREEN-27 self-report form (The KIDSCREEN Group Europe, 2006), the Emotion Regulation Questionnaire for Children and Adolescents (ERQ-CA; Gullone and Taffe, 2012), the Self Perception Profile for Children (SPPC; Harter, 1985) and the Cheek and Buss Shyness Scale (Cheek, 1983), were completed. Parent completed measures (including the KIDSCREEN-27 proxy report form) were administered to parents in a sealed envelope distributed by the school, to be returned to the school office for the attention of the researcher. Parents who did not return these forms in a timely manner to the school were also approached by email. These measures are described below; all measures were completed with pencil on paper.

The KIDSCREEN-27 (The KIDSCREEN Group Europe, 2006) is a tool to measure HRQoL in 8-to-18-year-olds. It has frequently been used as a proxy measure of wellbeing (Ravens-Sieberer et al., 2010; Lloyd and Emerson, 2016). It can be used with both healthy adolescents and those with additional needs, allowing adolescents' perspectives of wellbeing to be compared between clinical groups. The KIDSCREEN has previously been used with children and adolescents aged 8–18 with DLD (cf. Hubert-Dibon et al., 2016; Coales et al., 2019). The KIDSCREEN-27 includes 27 items from 5 dimensions: Physical Wellbeing, Psychological Wellbeing, Autonomy and Parent Relations, Social Support and Peers, and School Environment. Adolescents and one parent were asked to rate items on a 5-point Likert scale, evaluating each statement in the context of the past week. The items comprising each dimension result in an overall T score (M = 50, SD = 10), with higher scores indicating more positive HRQoL. This questionnaire was normed using a large population-based sample of children (*N* = 22,827) from over 13 European countries (Ravens-Sieberer et al., 2007). In the current study, the Cronbach's alpha values of the child self-report KIDSCREEN-27 was 0.83, and the Cronbach's alpha values of the proxy (parent) report KIDSCREEN-27 was 0.85.

The ERQ-CA (Gullone and Taffe, 2012) provided a measure of participants' emotion regulation strategies in terms of “Cognitive Reappraisal” (CR; i.e., reshaping how one thinks about certain situations so that they take less of an emotional toll) and “Expressive Suppression” (ES; i.e., attempting to hide, inhibit or reduce ongoing emotion-expressive behavior). Each item consists of a statement, e.g., “I keep my emotions to myself”; participants attributed a number from 1 (strongly disagree) to 5 (strongly agree) to each. For both CR and ES dimensions, a total score was computed by summing relevant items. Gullone and Taffe (2012) found the alpha reliability coefficients to range from 0.82 to 0.85 for the 6-item CR scale and from 0.69 to 0.79 for the 4-item ES scale. The Cronbach's alpha of the ERQ-CA in the current study was 0.81 for CR and 0.46 for ES. Due to this low alpha value for the ES scale, ES was not used as a variable in this study.

The Social Competence subscale of the SPPC (Harter, 1985) was used to assess participants' self-perceived social competence. This subscale contains six items and each item consists of two opposite descriptions. Participants choose a description and indicate whether it is somewhat true or very true for them. Each item is scored on a four-point scale (higher scores reflect a more positive view of oneself) and a total score is computed by summing items. The Cronbach's alpha of the SPPC in the current study was 0.92.

The Cheek and Buss Shyness Scale (Cheek, 1983) was used to assess sociability. The scale has 13 items, with responses from 1 (very untrue) to 5 (very true), requiring the respondent to indicate how much he or she wants to be/interact with people. Example items include: “I like to be with people,” and “I prefer working with others rather than alone.” A total Sociability score was computed by summing the items. The Cronbach's alpha of the Chuck and Buss scale in the current study was 0.85.

## Results

### Comparisons of Wellbeing

**Figure 1:**
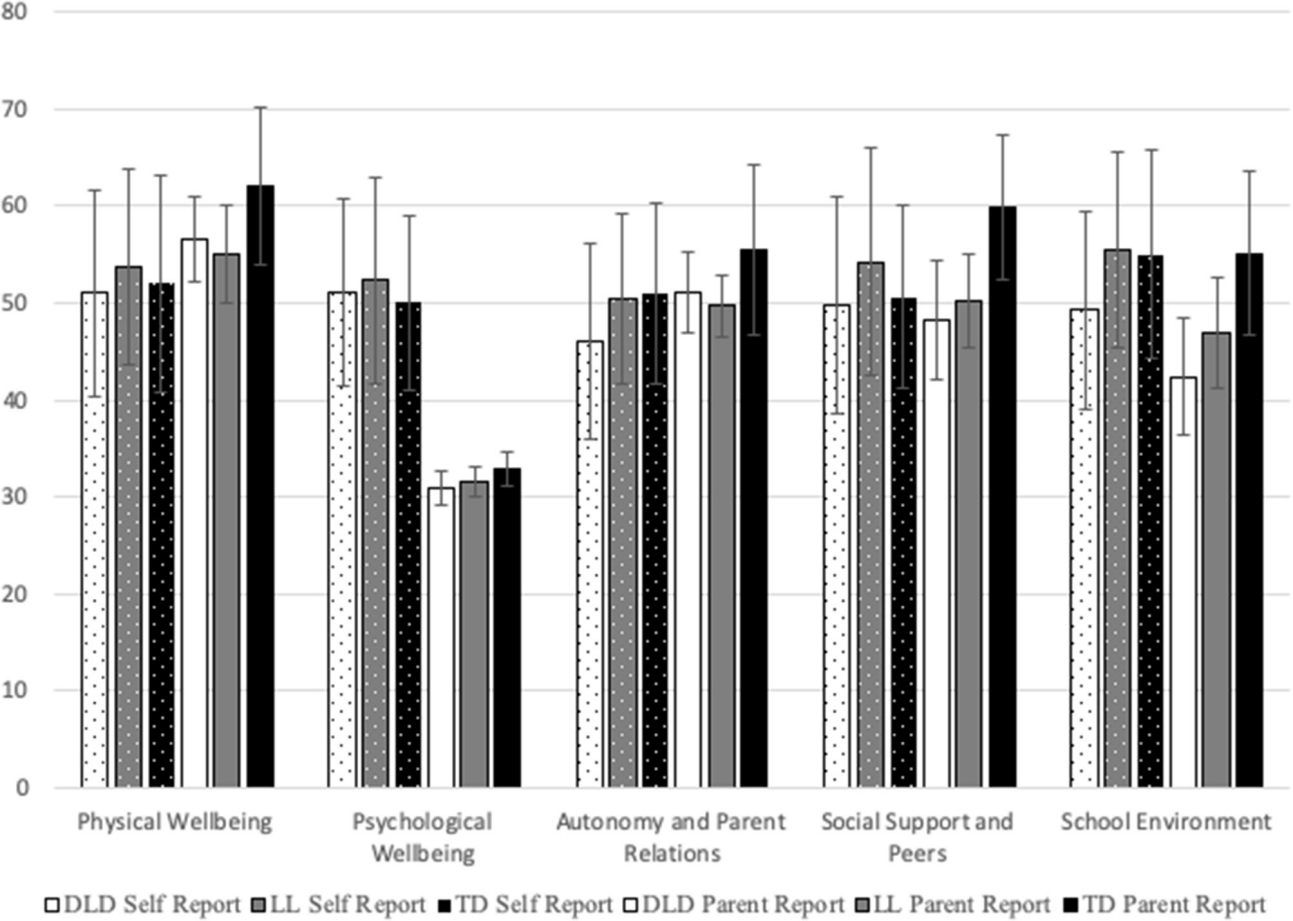
Mean T-scores across each dimension of the KIDSCREEN-27 according to self and parental report. Error bars reflect standard deviation.

Wellbeing as measured by self (adolescent) reported and proxy (parent) reported KIDSCREEN-27 questionnaires were explored by group. Adolescent report scores fell within one standard deviation of the European norming sample of children aged 8–11 for all five dimensions for all groups (Ravens-Sieberer et al., 2007). Parental report scores also fell within one standard deviation of the norming sample for all groups on four of the five dimensions; Parental reports of Psychological Wellbeing were below European norms for all three groups. For mean T-Scores across each dimension of the KIDSCREEN-27 according to self and parental report, please see Figure 1.

Further examination of the dimensions of the KIDSCREEN determined that Adolescent reported wellbeing did not significantly differ by group for Physical Wellbeing, Psychological Wellbeing or Social Support and Peers. However, there was a trend for group differences in Autonomy and Parental Relations (*p* = 0.064, $\eta_{\text{p}}^{\;2}$ = 0.05), and *post hoc* tests confirmed that adolescents with DLD had lower self-reported Autonomy and Parental Relations than their TD peers. The DLD group had significantly lower School Environment scores than their LL and TD peers. For statistics by group, please see Table 2.

**Table 2:** KIDSCREEN-27 adolescent and parent report T scores by group.

**KIDSCREEN-27 Dimension**	**Developmental Language Disorder mean (SD) (range) *n* = 30**	**Low Language mean (SD) (range) *n* = 29**	**Typically Developing mean (SD) (range) *n* = 48**	**Test statistics**	**European norming data mean (SD) *n* = 5,142–5,905**
**Adolescent report**					
Physical wellbeing	51.09^a^ (10.63) (32.69–73.20)	53.67^a^ (10.12) (36.55–73.20)	52.00^a^ (11.17) (25.07–73.20)	*F*_(2, 106)_ = 0.44, *p* = 0.646, $\eta_{\text{p}}^{\;2}$ = 0.01	53.72 (9.96)
Psychological wellbeing	51.08^a^ (9.73) (41.75–73.53)	52.35^a^ (10.71) (40.39–73.53)	50.03^a^ (9.06) (29.42–73.53)	*F*_(2, 106)_ = 0.52, *p* = 0.599, $\eta_{\text{p}}^{\;2}$ = 0.01	53.04 (9.94)
Autonomy and parent relations	46.06^a^ (10.07) (21.39–74.39)	50.47^a^^,^^b^ (8.83) (34.69–74.39)	51.07^b^ (9.36) (30.31–74.39)	*F*_(2, 106)_ = 2.82, *p* = 0.064, $\eta_{\text{p}}^{\;2}$ = 0.05	51.57 (10.32)
Social support and peers	49.84^a^ (11.22) (19.37–66.34)	54.28^a^ (11.82) (29.32–66.34)	50.64^a^ (9.36) (26.73–66.34)	*F*_(2, 106)_ = 1.52, *p* = 0.224, $\eta_{\text{p}}^{\;2}$ = 0.03	51.00 (10.04)
School environment	49.27^a^ (10.15) (27.81–71)	55.56^b^ (10.13) (38.68–71.00)	55.00^b^ (10.72) (30.55–71.00)	*F*_(2, 106)_ = 3.55, *p* = 0.032, $\eta_{\text{p}}^{\;2}$ = 0.06	54.03 (10.36)
**Parental report**					
Physical wellbeing	56.52^a^ (4.36) (49.54–63.68)	55.07^a^ (5.05) (49.54–63.68)	62.06^b^ (8.10) (49.54–71.23)	*F*_(2, 106)_ = 12.74, *p* < 0.001, $\eta_{\text{p}}^{\;2}$ = 0.20	52.65 (9.49)
Psychological wellbeing	30.90^a^ (1.83) (26.61–32.99)	31.61^a^ (1.61) (28.20–36.38)	32.91^b^ (1.74) (29.78–34.66)	*F*_(2, 106)_ = 13.40, *p* < 0.001, $\eta_{\text{p}}^{\;2}$ = 0.21	51.72 (9.57)
Autonomy and parent relations	51.11^a^ (4.19) (42.18–62.95)	49.73^a^ (3.21) (43.79–56.01)	55.54^b^ (8.75) (47.22–68.41)	*F*_(2, 106)_ = 8.51, *p* < 0.001, $\eta_{\text{p}}^{\;2}$ = 0.14	50.76 (9.66)
Social support and peers	48.23^a^ (6.11) (39.97–59.67)	50.16^a^ (4.81) (39.97–59.67)	59.90^b^ (7.48) (52.59–70.34)	*F*_(2, 106)_ = 36.92, *p* < 0.001, $\eta_{\text{p}}^{\;2}$ = 0.42	50.44 (9.39)
School environment	42.37^a^ (6.06) (32.95–59.34)	46.97^b^ (5.68) (32.95–59.34)	55.15^c^ (8.47) (47.69–70.67)	*F*_(2, 106)_ = 31.57, *p* < 0.001, $\eta_{\text{p}}^{\;2}$ = 0.38	52.95 (9.86)

a,b,c *Values with the same superscript do not differ when p < 0.05*.

In contrast, for parental report, the DLD and LL group were considered to have lower wellbeing on all five dimensions of the KIDSCREEN relative to their TD peers. The DLD and LL group only differed for the dimension of School Environment, where the LL group received a higher rating (see Table 2).

### Consistency of Parent vs. Adolescent Reported Wellbeing

Paired sample *T*-test analyses were conducted to explore the relationship between parent and adolescent reported wellbeing by group. The DLD group parent and child reported wellbeing scores were found to be significantly different for all domains except Social Support and Peers. The LL group parent and child reported wellbeing scores were found to be significantly different for Psychological Wellbeing and School Environment. The TD group parent and child reported wellbeing scores were found to be significantly different for all domains except School Environment. Please see Table 3 for details.

**Table 3:** Paired sample *T*-test analysis of child and parent reported wellbeing by group.

**Dimension of the KIDSCREEN-27**	**Developmental Language Disorder *n* = 30**	**Low Language *n* = 29**	**Typically Developing *n* = 48**
Physical wellbeing	*t*_(29)_ = −2.43, *p* = 0.022, d = 0.44, 95% CI (−10.00 to −0.86)	*t*_(28)_ = −0.66, *p* = 0.513, d = 0.12, 95% CI (−5.72 to 2.92)	*t*_(47)_ = −5.50, *p* < 0.001, d = 0.79, 95% CI (−13.73 to −6.38)
Psychological wellbeing	*t*_(29)_ = 11.42, *p* < 0.001, d = 2.08, 95% CI (16.56 to 23.79)	*t*_(28)_ = 10.23, *p* < 0.001, d = 1.90, 95% CI (16.58 to 24.89)	*t*_(47)_ = 12.91, *p* < 0.001, d = 1.86, 95% CI (14.45 to 19.79)
Autonomy and parent relations	*t*_(29)_ = −2.52, *p* = 0.017, d = 0.46, 95% CI (−9.15 to −0.96)	*t*_(28)_ = 0.38, *p* = 0.704, d = 0.07, 95% CI (−3.19 to 4.66)	*t*_(47)_ = −2.46, *p* = 0.018, d = 0.36, 95% CI (−8.13 to −0.81)
Social support and peers	*t*_(29)_ = 0.73, *p* = 0.472, d = 0.13, 95% CI (−2.92 to 6.14)	*t*_(28)_ = 1.94, *p* = 0.063, d = 0.36, 95% CI (−0.23 to 8.47)	*t*_(47)_ = −5.35, *p* < 0.001, d = 0.77, 95% CI (−12.74 to −5.77)
School environment	*t*_(29)_ = 3.56, *p* = 0.001, d = 0.65, 95% CI (2.93 to 10.86)	*t*_(28)_ = 3.82, *p* < 0.001, d = 0.71, 95% CI (3.98 to 13.21)	*t*_(47)_ = −0.85, *p* = 0.933, d = 0.01, 95% CI (−3.81 to 3.51)

### Difference Between Parent and Adolescent Reports – Report Agreement Scores

To explore parental and self report consistency further, a “report agreement” score was created by dividing the parent rating by the adolescent rating (cf. Norbury and Bishop, 2002; Lucas and Norbury, 2015; Gough Kenyon et al., 2018). Thus, a “report agreement” score of 1 would reflect complete agreement between parent and adolescent rating, without differences in scores being skewed by proportional differences. Concordantly, the greater the distance from 1, the lower agreement reflected by the report agreement score. Scores > 1 reflect higher parent ratings than adolescent ratings; scores < 1 reflect higher adolescent ratings than parent ratings.

For the dimension of Physical Wellbeing, the TD group had the lowest reported agreement, which significantly differed from the LL group, but not the DLD group. In contrast, for Psychological Wellbeing the DLD group had the lowest report agreement, which significantly differed from the TD group, but not the LL group. For Autonomy and Parental Relations both the TD and DLD groups had greater discrepancy than the LL group. For Social Support and Peers the TD group had the lowest report consistency, whereas for the School Environment both the DLD and LL group has lower report consistency than the TD group. Please see Table 4 for details of the report agreement score of each dimension of the KIDSCREEN-27, by group.

**Table 4:** Report agreement score of parent and adolescent reported wellbeing by group.

**Variable**	**Developmental Language Disorder mean (SD) *n* = 30**	**Low Language mean (SD) *n* = 29**	**Typically Developing mean (SD) *n* = 48**	***Test statistics***
Physical wellbeing	1.15^a^^,^^b^ (0.25)	1.06^b^ (0.23)	1.25^a^ (0.31)	*F*_(2, 106)_ = 4.00, *p* = 0.021, $\eta_{\text{p}}^{\;2}$ = 0.01
Psychological wellbeing	0.62^a^ (0.11)	0.63^a^^,^^b^ (0.12)	0.68^b^ (0.13)	*F*_(2, 106)_ = 2.72, *p* = 0.071, $\eta_{\text{p}}^{\;2}$ = 0.05
Autonomy and parent relations	1.16^a^ (0.31)	1.02^b^ (0.19)	1.12^a^ (0.27)	*F*_(2, 106)_ = 2.55, *p* = 0.083, $\eta_{\text{p}}^{\;2}$ = 0.05
Social support and peers	1.03^a^ (0.31)	0.97^a^ (0.26)	1.23^b^ (0.31)	*F*_(2, 106)_ = 8.08, *p* = 0.001, $\eta_{\text{p}}^{\;2}$ = 0.13
School environment	0.89^a^ (0.20)	0.88^a^ (0.20)	1.04^b^ (0.24)	*F*_(2, 106)_ = 6.69, *p* = 0.002, $\eta_{\text{p}}^{\;2}$ = 0.11

a,b,c *Values with the same superscript do not differ when p < 0.05*.

### Predictors of Parent and Adolescent Report Consistency in Psychological Wellbeing

For the dimension of Psychological Wellbeing, regression analysis was conducted to explore predictors of variance by group with three predictor variables: cognitive reappraisal, social competence and sociability. Please see Table 5 for description of these predictor variables by group.

**Table 5:** Predictor variables: cognitive reappraisal, social competence and sociability by group.

**Variable**	**Developmental Language Disorder mean (SD) *n* = 30**	**Low Language mean (SD) *n* = 29**	**Typically Developing mean (SD) *n* = 48**	***Test statistics***
Emotional Regulation: Cognitive Reappraisal	20.00^a^ (4.53)	22.31^a^ (4.425)	21.21^a^ (5.04)	*F*_(2, 106)_ = 1.75, *p* = 0.178, $\eta_{\text{p}}^{\;2}$ = 0.03.
SPPC Social Competence	14.02^a^ (5.07)	11.66^a^ (4.44)	12.41^a^ (3.98)	*F*_(2, 106)_ = 2.23, *p* = 0.113, $\eta_{\text{p}}^{\;2}$ = 0.04
Sociability	36.53^a^^,^^b^ (5.25)	37.52^a^ (4.85)	35.38^b^ (4.25)	*F*_(2, 106)_ = 1.93, *p* = 0.150, $\eta_{\text{p}}^{\;2}$ = 0.04

Regression analysis found that predictors differed by group. For the DLD group the total model was not significant, *F*_(3, 29)_ = 2.06, *p* = 0.131, $\eta_{\text{p}}^{\;2}$ = 0.19, although social competence was a significant predictor (*p* = 0.033). For the LL group the model was significant, *F*_(3, 28)_ = 4.00, *p* = 0.019, $\eta_{\text{p}}^{\;2}$ = 0.32, and explained 32% of the variance in report agreement in Psychological Wellbeing. Cognitive reappraisal and sociability were significant predictors (both *p* < 0.05). For the TD group, the model was significant, *F*_(3, 47)_ = 4.39, *p* = 0.009, $\eta_{\text{p}}^{\;2}$ = 0.23, and explained 23% of the variance in report agreement in Psychological Wellbeing. Social competence was the sole significant predictor (*p* = 0.002). Please see Table 6 for further details.

**Table 6:** Regression analysis predicting report agreement score in psychological wellbeing by group.

	**β**	***t***	***p***	**95% CI**	**Zero-order correlation**	**Semi-partial correlation**
**DLD Group**						
Cognitive reappraisal	−0.17	−0.92	0.368	(−0.01 to 0.01)	−0.17	−0.16
Social competence^*^	0.40	2.26	0.033	(0.01 to 0.02)	0.41	0.40
Sociability	0.04	0.22	0.829	(−0.01 to 0.01)	0.04	0.04
**LL Group**						
Cognitive reappraisal^*^	−0.38	−2.15	0.041	(−0.02 to 0.00)	−0.37	−0.35
Social competence	0.04	0.24	0.810	(−0.01 to 0.01)	0.23	0.04
Sociability^*^	0.43	2.59	0.016	(0.01 to 0.02)	0.40	0.43
**TD Group**						
Cognitive reappraisal	−0.02	−0.13	0.896	(−0.01 to 0.01)	−0.12	−0.02
Social competence^*^	0.48	3.38	0.002	(0.01 to 0.03)	0.43	0.45
Sociability	−0.23	−1.66	0.104	(−0.02 to 0.01)	−0.11	−0.22

* *Significant when p < 0.05*.

## Discussion

This study contributes to the limited extant body of work by providing both adolescent and parent reported wellbeing in a sample of adolescents with DLD, adolescents with LL, and their TD peers. The findings indicated that adolescents with DLD and LL have more similar wellbeing to their TD peers when assessed via self-report than via parental report. Parent and adolescent reported wellbeing scores differ across multiple dimensions of wellbeing, regardless of the adolescent's language ability. This difference is particularly striking in Psychological Wellbeing; parent reports indicate significantly lower levels of Psychological Wellbeing than the levels indicated by their children's self-reports. Predictors of rater consistency of psychological wellbeing also differ between groups; whilst cognitive reappraisal and sociability predicted level of agreement for adolescents with LL, social competence predicted agreement in DLD and TD. This emphasizes the importance of including the full spectrum of need when investigating the impact of language ability on wellbeing, including consistency between self and proxy reported wellbeing.

### Group Comparisons of Wellbeing

It was predicted that there would be few group differences in wellbeing according to self-report. Consistently, adolescent ratings of wellbeing were similar across groups on three of the five wellbeing dimensions, namely, Physical Wellbeing, Psychological Wellbeing and Social Support and Peers. However, those with DLD had lower self-reported Autonomy and Parental Relations than their TD peers, and both the DLD and LL group had lower School Environment scores than their TD peers. Parental reports of wellbeing were also consistent with the study's first hypothesis; those for TD adolescents were significantly higher than those for adolescents with DLD and LL, across all five domains.

The adolescent rated findings are reminiscent of Arkkila et al. (2009, 2011), who found self-reported wellbeing in 8–11 year olds (2011) and 12–16 year olds (2009) not to significantly differ between DLD and TD groups. Recently, Coales et al. (2019) also found that children and adolescents with DLD (aged 7–13) largely reported wellbeing levels within normative ranges. Unlike the current study, they did not find the DLD group had lower wellbeing on Autonomy and Parental Relations and School Environment but instead that they had lower scores for Moods and Emotions and the Social Acceptance/Bullying domains, which were not included in the shorter version of the KIDSCREEN used in the current study. Perhaps this difference may be attributable to the differential ages of the study participants; this hypothesis warrants further investigation. The parental ratings of wellbeing agree with recent comparisons between parents of TD children (aged 8–18) and their peers with DLD, with parental ratings being significantly lower for their children with DLD across multiple measures (c.f. Van Agt et al., 2011; Hubert-Dibon et al., 2016; Eadie et al., 2018). The contrast between adolescent and parent reported wellbeing across groups highlights the importance of investigating the difference between the two.

### Consistency of Adolescent vs. Parent Reported Wellbeing

It was predicted that there would be significant differences between parent and adolescent ratings of wellbeing, as is the case for youth with ASD (cf. Potvin et al., 2015; Egilson et al., 2017), and as has been found between tutor and adolescent ratings in DLD (Valera-Pozo et al., 2020). The findings strikingly support this hypothesis; there were significant differences across multiple well-being dimensions, for each of the three groups. Examination of effect sizes indicated that the difference between parent and adolescent report was greatest for all three groups for Psychological Wellbeing. Interestingly, for all three groups, parents rated their child's psychological wellbeing lower than the adolescent's own perception. Importantly, this pattern was not replicated for all KIDSCREEN-27 dimensions; for Physical Wellbeing and Autonomy and Parent Relations parent ratings were higher than that of the adolescents' for both DLD and TD groups, although there was no significant difference for the LL group. For Social Support and Peers, there was a significant difference between the self-reported wellbeing of TD adolescents and the much higher levels of wellbeing reported by their parents, with no significant difference found between those with DLD and LL and their parents. Oppositely, for School Environment, parents of those with DLD and LL significantly underestimated the wellbeing levels self-reported by their children, while the parents of TD adolescents did not differ from their children's reports.

It has been observed that the level of variance between parent and adolescent measures is directly linked to the visibility of the dimension to parents (Verrips et al., 2000). While parents of adolescents have limited access to their adolescents' experiences and social relationships via observation or correspondence with the school, they cannot gain insight into their children's assessment of their own emotional states, beyond what can be gleaned by information the adolescent offers, or what emotions they outwardly portray. There is a possibility that parents of TD adolescents worry less about psychological, social and school domains compared to parents of adolescents with DLD and LL (Hughes et al., 2009; Hubert-Dibon et al., 2016; Eadie et al., 2018). Nonetheless, parental perspective is a valuable resource and this study importantly includes both parent and adolescent ratings, with no opportunity cost.

### Predictors of Parent and Adolescent Report Consistency in Psychological Wellbeing

As expected, predictors of consistency between parent and adolescent ratings of Psychological Wellbeing differed between groups. It was anticipated that similarities would emerge between DLD and LL groups, with TD adolescents showing a different profile of predictors. However, adolescents with TD and DLD showed similar profiles, with both groups levels of agreement with their parents ratings being predicted by their self-perceived social competence. Contrastingly, the agreement between adolescents with LL and their parents was predicted by levels of cognitive reappraisal strategy use and sociability. The paucity of information available concerning either the wellbeing of adolescents with LL or the agreement between parental and adolescent perspectives for this group makes it difficult to ascertain the reason for this difference. However, we know that while adolescents with LL are at a similar risk of negative outcomes due to language impairment as their peers with DLD (Conti-Ramsden et al., 2017) their lack of diagnosis means no entitlement to the support that a child with a diagnosis would be entitled to Gough Kenyon et al. (2018). These adolescents may employ different coping strategies, thus resulting in communication with parents being affected by different factors.

### Study Evaluation

This study makes a valuable contribution to the extant literature on the wellbeing of adolescents with DLD, LL, and TD. While this study provides insight into the differences between groups in terms of parental vs. adolescent perspective, more research is necessary to facilitate understanding of why some of these differences are present. For example, the similar profile of predictors for parent-adolescent agreement for TD adolescents and their peers with DLD in contrast to adolescents with LL ability (a group who, in terms of language ability, are ostensibly the midway point between DLD and TD) warrants further exploration. Inclusion of qualitative measures that may elicit fuller responses from parents and adolescents may be a valuable step toward understanding.

The inclusion of a LL group is a particular strength of this study. No study has heretofore included adolescents with LL in an exploration of the relationship between language profile and wellbeing. It is clear that this group is not homogeneous with either the TD or DLD groups and warrants inclusion as a group in its own right. The contrast between parental and adolescent report of School Environment is particularly striking; according to parental report, wellbeing in this area for adolescents with LL is significantly higher than their peers with DLD and significantly lower than their TD peers. However, adolescent reported scores of School Environment show LL scores to be very similar to TD scores, and significantly greater than reports of adolescents with DLD. This dimension explores the child's perception of their cognitive capacity, learning, concentration, feelings about school and relationship with teachers. It could be that children with LL may not be communicating the positive aspects of their school life to their parent, despite having a generally positive affect concerning school. Alternately, parents may be perceiving that their child's language impairment is having academic consequences (i.e., a halo effect). This has yet to be examined.

Importantly the study sample comprised a much narrower age range than many of the other studies in this area (Records et al., 1992; Johnson et al., 2010; Conti-Ramsden et al., 2016; Hubert-Dibon et al., 2016; Coales et al., 2019), enabling conclusions to be drawn about a more specific demographic group at a particular developmental point.

A notable limitation of this study is the fact that the KIDSCREEN-27 parent and self-reports were not completed at the same time by parents and adolescents. For some participants, those whose parents completed the proxy reports and returned these forms promptly, there was little time difference. For others, there was a considerable time difference. However, a partial correlation analysis was conducted to explore the moderating effect of the time difference between completion dates of adolescent and parent reports on the strength of the correlation between adolescent and parent reports. Time was not found to be impactful, with correlations remaining non-significant for all dimensions for all three groups (all *p* > 0.09).

### Educational and Clinical Implications

The results of this study have favorable implications. While it is important to acknowledge that the sample for this study were drawn from mainstream schools, and therefore are not representative of adolescents with minimal language skill, the findings for the spectrum of language ability included are extremely positive. The self-reported wellbeing of those with DLD and LL in this study are very close to the population norms. The lowest scores were reported in the domain of Autonomy and Parent Relations so interventions targeting wellbeing in these populations would be well advised to prioritize this area.

The findings of this study also provide further confirmation of the evidence that adolescents with neurodevelopmental disorders, and DLD in particular, can complete questionnaires and are capable of reporting their own experience (cf. Owen et al., 2004; Palikara et al., 2009). The importance of the perspective of under 18s has been highlighted in international (UNICEF, 1999) and UK (HMSO, 1989) policy. It is especially important to account for the adolescents voice when we consider the further substantiated evidence that this perspective differs so significantly from their parents.

The difference between parent and adolescent reported wellbeing is greatest in terms of Psychological Wellbeing. The implications of this are distinct for adults interacting with adolescents with DLD and LL vs. TD peers. For the former, parents perceive psychological wellbeing to be an area of concern for their adolescents. The reality is that their children do not deem themselves to be disproportionately suffering in this domain; a finding that is very positive for alleviating undue parental worry. On the other hand, it would appear that parents can overestimate the robustness of the Psychological Wellbeing of their TD adolescents. The vulnerabilities of all adolescents, including TD adolescents, must be appreciated when planning educational interventions and this group must be included.

## Conclusion

This study explored the wellbeing of adolescents with DLD, adolescents with LL proficiency and their TD peers. It contributes to the limited extant body of work examining wellbeing in DLD and LL by providing both parent and adolescent rated measures of wellbeing. Ultimately, it emphasizes the necessity of allowing adolescents to report their own wellbeing, as their perspective is likely to differ from their parents, particularly in terms of their Psychological Wellbeing. The degree of variance between adolescents and their parents can be predicted by cognitive reappraisal and sociability for adolescents with LL while social competence predicts the level of agreement in DLD and TD. Interventions designed to increase wellbeing in adolescents should incorporate the full spectrum of language abilities and acknowledge the limited insight parents may have into their adolescents' experience.

## Data Availability Statement

The raw data supporting the conclusions of this article will be made available by the authors, without undue reservation.

## Ethics Statement

The studies involving human participants were reviewed and approved by The Research Ethics Committee of the University of Roehampton, London. Written informed consent to participate in this study was provided by the participants' legal guardian/next of kin.

## Author Contributions

SG, OP, and RL: conceptualization and writing—review and editing. SG: formal analysis, investigation, data curation, writing—original draft preparation, and project administration. OP and RL: supervision. All authors contributed to the article and approved the submitted version.

## Conflict of Interest

The authors declare that the research was conducted in the absence of any commercial or financial relationships that could be construed as a potential conflict of interest.
